# Women's experiences of VBAC in Cyprus: a qualitative study

**DOI:** 10.1186/s12884-021-04193-7

**Published:** 2021-11-12

**Authors:** Eleni Hadjigeorgiou, Constantina Katsie, Maria Papadopoulou, Maria Dolores Christofi, Andri Christoforou

**Affiliations:** 1 Department of Nursing, School of Health Sciences, Cyprus University of Technology, 15, Vragadinou Str, 3041 Limassol, Cyprus; 2 Department of Nursing, School of Health Science, Limassol, Cyprus; 3 Department of Social and Behavioral Sciences, European University Cyprus, Nicosia, Cyprus

**Keywords:** VBAC, vaginal birth after c-sections OR vaginal birth after caesarean section, women’s views, women’s experiences, women’s perceptions

## Abstract

**Background and aim:**

In 21st century, there has been an increasing interest in vaginal birth after previous caesarean section (VBAC) in Cyprus, a country with a very high operative birth rate. Research-based evidence of women’s VBAC experiences in Cyprus is non-existent, despite its significance for the well-being of mothers and families. The aim of this study is to gain insight into the women’s lived experience of VBAC in Cyprus. In this study women’s experiences of VBAC are explored for the first time in Cyprus.

**Method:**

The study is qualitative and exploratory in nature. Data were collected through semi-structured interviews with 10 women, who experienced vaginal birth after a previous caesarean section (VBAC) in Cyprus. A descriptive phenomenological approach was employed for the analysis of data.

**Results:**

Analysis of data yielded four major themes: (a) medicalization of childbirth, (b) preparing for a VBAC, (c) birth environment, and (d) healing through VBAC. On the whole, the women interviewed described their previous experience of CS as traumatic, in contrast to vaginal childbirth. VBAC was considered an utterly positive experience that made the women feel empowered and proud of themselves.

**Conclusion:**

This study offers valuable insight into a newly researched subject in Cyprus, which is necessary for advancing perinatal care in Cyprus. The findings indicate that women need evidence-based information, guidelines on birthing options, good preparation with tailored information and personalized care for a successful vaginal birth after a previous caesarean section. Proper, non-biased, consultations are a main factor that affects women’s choice of mode of birth. The introduction of new, women-friendly perinatal strategies that respect and promote childbirth rights is imperative in the case of Cyprus. All women have the right to exercise informed choice and the choice to alternative birthing options.

## Background

In the 21^st^ century there has been a global concern over the increase in the number of caesarean sections (CSs) as these have been associated with an increased perinatal mortality and morbidity [1]. The observed increase constitutes an important public health issue, with potential negative consequences on the health of both the mother and the newborn [2]. While WHO recommends the ideal rate of CSs to be between 10-15% of all births [3], the CS rate has been increasing worldwide, with some countries exceeding 40% [1]. For example, the CS rate reached 58.1% in Dominican Republic, 55.5% in Brazil, 55.5% in Egypt and 53.1% in Turkey in 2015 [4]. Although a CS is considered a necessary life-saving surgery for the health of the mother and newborn when medically indicated [5], many women who give birth by CS report negative psychosocial effects including grief, a sense of failure, a sense of a loss of control, and feeling betrayed by those that cared for them [6, 7]. CS after a previous CS is the most common and the main factor of increased rates of caesarean sections [8, 9].

VBAC has numerous benefits including the avoidance of operative complications, shorter hospitalizations, better maternal satisfaction, as well as reduced maternal morbidity and mortality rates [10]. The American College of Obstetricians and Gynecologists [11] has reported that VBAC is a safer alternative to repeated CS for many women and should be recommended accordingly. In the United States, The VBAC rate increased by 7% for the period 2016–2018 and for women in their 20s and 30s, but failed, nevertheless, to meet the federal government’s Healthy People 2020 target of 18.3% [12]. In the European Union, some countries such as Finland, Netherlands and Sweden have high VBAC rates, ranging from 45 to 55%. Yet, other countries such as Germany, Ireland and Italy present significantly lower rates, ranging from 29 to 36% [9, 13].

While in-depth examinations of women’s VBAC views and experiences is limited [7–9], there are certain common themes deriving from the few qualitative studies available. For example, a meta-synthesis of women’s experiences in the Anglo-American context showed that navigating option of VBAC was like “groping through the fog” [7]. The women perceived the information available from the healthcare system and professionals as both unclear and contrasting, often resulting in ambivalent decision-making. The need for balanced, unbiased, and evidence-based information, as well as the need for a more supportive attitude from clinicians was also evident in other contexts, even in countries with high VBAC rates and a birth culture that promotes VBAC such as Finland, the Netherlands, and Sweden [9]. Making informed decisions and having a reliable and trustworthy collaboration with professionals is of utmost importance to women and this is highlighted throughout the literature [7–9, 14]. Another common finding is that the women who were strongly determined to birth vaginally after a CS viewed birth as a significant life event; vaginal birth had a personal meaning for women, contributing to their determination to achieve VBAC [7–9, 14–17]. Giving birth vaginally after CS is often described by women as an empowering experience [7, 9], as a miraculous and spiritual experience that strengthens their sense of motherhood, and consequently the mother-baby bonding [18]. Overall, the literature suggests that women’s decision-making about vaginal birth after caesarean is influenced by multiple and diverse factors including medical, psychological, material and socio-cultural parameters. The need for more qualitative research, and particularly research conducted in different socio-cultural contexts and maternity settings, is emphasized consistently throughout the literature.

### CS and VBAC in Cyprus^1^

In 2018, there were 9406 live births and 29 fetal deaths in Cyprus, giving a fetal mortality rate equal to 3.1 per 1000 total births. Women less than 20 years old accounted for 2.3% of births, women aged 20-34 for 72.6%, and women aged 35 and above for 25.1%. On average, 5% of births are of women who became pregnant after some form of assisted reproductive technique [13]. Childbirth is a highly medicalized experience as shown by the extremely high CS rates [19]. The latest figures show that in 2018, Cyprus had the highest rate of caesarean deliveries out of 31 European countries, with 53.6% of babies born this way [13]. Caesarean sections were slightly more common in the private sector (54.9%) compared with public hospitals (51.1%).

Up until June 2019, when a national health insurance scheme was introduced, public hospitals were financed by the government and were accessible to civil servants and to people meeting the criteria of specific socio-economic backgrounds. In the private sector, the patients had to pay themselves or were covered by private health insurance [20]. The primary caregivers of antenatal, intrapartum, and postnatal care in Cyprus are obstetricians/ gynecologists and midwives. Community midwifery is not available, while home births are prohibited by law. There is currently only one midwife-led unit, which can accommodate one or two women at a time. Under the new General Healthcare System (GESY), which aims to provide affordable and effective medical care to all Cyprus residents, women have the right to choose any obstetrician who is associated with the scheme. Midwives participate in the system as independent healthcare providers and pregnant women/mothers can have direct contact with them up to 6 times [21].

In 2017, the non-governmental organisation “Birth Forward” held a public awareness campaign, advocating for the decrease of unnecessary CSs. At the same time, the Ministry of Health designated a committee to tackle the issue and to promote vaginal birth [19]. The campaign stirred up some controversy and eventually led to a public dialogue on the country’s high CS rates, for the first time. The various stakeholders involved (i.e. women, activists, midwives, OB/GYNs and the state) provided diverse and multiple factors that could potentially account for the observed rates of CS [19]. The Midwives Association and women’s activist groups argued that the main reason for the high rates of CS was the lack of informed choice for childbearing women; choosing between CS and vaginal birth can be difficult as the benefits and risks are not discussed openly and publicly. The fear of the unknown and the fear of birth itself could also contribute to the high rates of maternal requests for CS. The unethical behavior of some obstetricians was also a central theme before the introduction of GESY, with many women and journalists arguing that several obstetricians led women to opt for a CS for their own convenience, but perhaps more importantly, for financial reasons (i.e. at the time, the financial cost for vaginal and CS births was estimated around €800-1500 and €3500-6000, respectively). The changing landscape of reproduction trends (i.e. the introduction and the increasing use of scientifically advanced assisted reproduction techniques resulting in multiple births, and the increasing age of women at childbirth) was also proposed as a reason for the increasing CS rates; this argument was mainly supported by OB/GYNs and state representatives [19].

VBAC remains a controversial topic, while there are no national guidelines or specific targets to be achieved. Women in both the public and the private sectors are often advised by their obstetricians that a CS after a previous CS is in the best interest of the baby and most women succumb to this recommendation [19]. As only a handful of obstetricians offer evidence-based information about VBAC through pre-birth seminars, women, at large, are not presented with balanced and unbiased information that would enable them to make informed decisions.

Many women do not have a choice, either due to institutional practice restrictions or because of healthcare providers who cannot or do not offer VBAC as an option.

Overall, there is currently no data or research about VBAC practices or women’s experiences of VBAC in Cyprus. Interestingly, the lack of research on the topic has been highlighted in other contexts where research examining the psychosocial aspects of VBAC is minimal [22]. The few qualitative studies focusing on the experience of VBAC from women’s perspective illustrate that by and large, women make healthcare decisions based on incomplete and biased information regarding the risks and benefits associated with VBAC [23, 24]. This study will serve to add to the body of knowledge regarding VBAC from women's perspective.

## Method

### Study design

A qualitative study design was used in this study, influenced by the phenomenological methodology approach. Phenomenology uses two main approaches: descriptive, developed by Edmund Husserl and interpretive, developed by Martin Heidegger [25]. The descriptive (Husserlian) phenomenological qualitative approach is used as a method to “explore and describe the lived experience of individuals” [26], so it was deemed as the appropriate methodology for the aim of the study. This methodology acknowledges how individuals both within a social and within a personal world construct meanings. Within this framework, the researcher gets close to the participants’ personal worlds and investigates their views and interpretations, focusing on the understanding of an individual’s perspective as a whole.

### Sampling and data collection

Since this is the first study conducted in Cyprus to explore the issue, the authors were interested in interviewing women who managed to proceed with VBAC, so as to gain a more comprehensive understanding of their lived VBAC experience itself. While extending the sample to women who had sought VBAC but were not able to access or achieve it would undoubtedly offer invaluable insight into the emerging landscape of VBAC in Cyprus, this was beyond the focus of this initial, exploratory study. In order to raise awareness about the study quickly, snowball sampling was used to recruit participants through social media, and specifically through a local Facebook group whose members included mothers who have experienced VBAC. One of the researchers (C.K.) became a member of a group and after having sought permission from the administration of the group, uploaded an announcement about the study and the contact information (e-mail and telephone) of the researchers. The women who wished to participate in the study reached the researchers using this contact information.

Initially, the expected number of participants was based on previous relevant qualitative research, which included between four to thirty-five participants [7]. However, the numbers of participants in this study were caped at ten, based on the saturation of data, as there was no new information arising, and information occurred repeatedly when analyzing the transcripts. The inclusion criteria for the participation of the study participants were previous CS birth and a subsequent VBAC birth, birth to a healthy neonate in a public or private hospital maternity unit in Cyprus via VBAC, aged at least 18 years old at the time of the study, being able to speak and read Greek fluently and agreeing to participate in the study by signing an informed consent form. Women who had given birth more than 3 years prior to the interviewing phase were excluded from the study in order to avoid possible recall difficulties.

Data were collected from January 2020 to March 2020, through semi-structured interviews. The interviews took place at the participants’ preferred place, were recorded by the researcher and lasted on average about 50 minutes. The interview questions were developed by the authors (C.K., E.H.) on the basis of the relevant literature [1, 7–9, 14] and consisted of four main open-ended questions and clarifying questions about VBAC experience (Table 1). The interview approach was semi-structured in order to avoid limitations and biases towards specific themes and to open ‘gate’ to themes that the women wished to discuss during the interview.

**Table 1:** Interview guide

1.	How did you decide on vaginal birth after caesarean section delivery?
2.	How did the healthcare professionals react to your preferred childbirth mode? What kind of information did you receive?
3.	Could you tell us about your experiences with vaginal birth after caesarean section delivery?
4.	How would you describe your experience with vaginal birth after CS delivery?
	Further questions in order to clarify the participants responses: Please describe in as much detail as you can your experiences. Could you give an example? What do you mean by… ?

### Data analysis

All interviews were carefully transcribed verbatim, while listening to the recording several times to avoid missing out on important points. Thematic analysis of the transcripts was used as the method for data analysis, in order to abstract and isolate emerging themes from the interviews and group them into the main theme titles and subthemes (Table 2). To ensure the validity and reliability of the data, the transcription of each interview was sent to the each corresponding participant for confirmation. The data were analyzed simultaneously by two researchers (C.Κ., Ε.Η.) along the way and discussed on a constant basis. All data were analyzed and organized through open coding, while through the process of abstraction, emerging themes were identified and categorized into theme titles and sub-themes (C.Κ., Ε.Η.). In order to ensure validity of data, two other researchers (A.C., MD.C.) repeated the data analysis.

**Table 2:** Main themes and subthemes

Themes	Subthemes
Medicalization of childbirth	A culture of CSs Traumatic birth experience
Preparing for a VBAC	A need for clear answers Seeking information
Birth environment	Father’s involvement Family influences Interactions with health professionals
Healing through VBAC	Overcoming a negative experience with a positive one Fulfillment Bonding with baby

### Demographics

Eight out of ten women were Cypriot, one was Greek and one was German. The German mother lives permanently in Cyprus and speaks Greek fluently. The participants’ ages ranged from 28 to 38 years old. All participants were married and university-educated, 8 women were mothers to 2 children and 2 women were mothers to 3 children. Nine mothers gave birth in a private clinic and one at a public hospital. All women had a VBAC within the last 3 years.

### Ethics approval

Ethics approval was obtained by the Cyprus National Bioethics Committee (EEB 2019. 01. 62). This study has been conducted in accordance with the Declaration of Helsinki and all methods were performed in agreement with the relevant guidelines and regulations. The potential participants were fully informed via email about the aim, the type and the purpose of the study, as well as the method of data collection. They were also informed of their right to withdraw from the studyat any time and about the strategies that would be used to secure anonymity and confidentiality. All participants were aged above 18. Informed consent was obtained from all participants before the interviews and participants were allocated a code in order to keep their identities confidential according to GDPR guidelines.

## Results

The qualitative analysis of the women’s experiences of VBAC are presented in four themes and 10 sub themes as presented in Table 2.

### Medicalization of childbirth

The first theme that emerged from the analysis of data of the women’s experiences were factors reflecting on the women’s past experiences of childbirth and how that affected their decision-making and eventual choice to attempt VBAC. The theme is supported by two subthemes: (a) A culture of CSs and (b) traumatic birth experience. The women mentioned that the medicalization of childbirth is a big issue in Cyprus, as interventions during childbirth are considered the norm and cesarean section rates are constantly high. Amidst a “culture of CSs”, it is often difficult to find an obstetrician willing to offer the choice of VBAC.

### A culture of CSs

When referring to their experience with CS, six women were of the opinion that since there are no official guidelines published by the authorities about VBAC in Cyprus and no one moderating this within the healthcare system, most obstetricians find it easy to go by their preference and thus repeat a CS in the case of a previous CS. The women reported that they felt anxious as soon as they found out that they were pregnant because of this mentality. It was not uncommon for women to visit more than one obstetrician in an attempt to find an obstetrician who could help them to have a VBAC:

“*In Cyprus doctors prefer to do a CS after a CS. So, for me it was difficult to find an obstetrician who would offer me this choice*” (A1).

The women also described how the obstetricians eloquently explained to them the potential negative birth outcomes for their babies after a VBAC, which aimed to persuade them that a CS would be, after all, the best birthing option for them:

*“The doctor told to me that we are talking about permanent brain damage for your baby. I started crying and shaking”* (A1).

### Traumatic birth experience

The women recall their previous caesarean section as a traumatic birth that had negative psychological effects on them, accompanied by agony and disappointment. For some, the experience made them feel like a patient in a hospital:

“*And you know....you grab your suitcase and you go to the hospital healthy and well, but you leave feeling vulnerable and sick”*(A2).

Often, the women reported that they felt guilty and blamed themselves for not having been persistent enough to achieve a vaginal birth for their previous child. A woman, in her attempt to describe her experience, nervously smiled and said:

“*with my first baby, things didn't work out the way I wanted. But to tell you the truth, I didn't help myself enough to get what I wanted... [stops for a moment]. I didn't even go to childbirth classes for the first baby, I did nothing, I knew nothing*” (A9).

The antenatal preparation was an important issue for the mothers and its importance seemed to be greatly valued, since it was a source of valuable information for them.

### Preparing for a VBAC

The theme “Preparing for a VBAC” is connected to the women’s lived experiences of VBAC and it is analyzed into two interconnected subthemes: a) A need for clear answers, and b) Seeking for tailored information according to the mother’s needs. Having experienced CS as a traumatic experience led the women to seek more information and to demand for clear answers to their questions about the delivery of another baby. In contrast to their previous experience of childbirth, the women wanted to be well prepared for the birth of their next baby and to be able to exercise an informed choice regarding the mode of birth.

### A need for clear answers

Many women indicated that despite having asked many questions during their pregnancy, they often received confusing answers from their obstetricians. Some obstetricians refused to give them clear answers about the possibility of a VBAC. The need for personalized and tailored to their needs information, in order to be able to take an informed decision regarding the mode of childbirth, was emphasized. Healthcare professionals’ attitudes were an important part of maternity care, as the women wished to obtain information and guidance in good time in order to prepare themselves for the birth of their baby. Several women mentioned that they were not given or knew enough information during their first pregnancy and that affected their choice of birthing mode:

“*In my first pregnancy I did not have enough information, I did not know… that (the caesarean) would be a bad experience”* (A3).

Some women felt cheated and misinformed by their obstetricians when they (the obstetricians) claimed that they did not know what a VBAC is:

“*What is this thing - VBAC? I remember I left his office crying and upset”* (A1).

Another woman explained that her doctor’s behavior made her even more determined to seek on further information and to look for another doctor to take care of her:

“*I said to myself: What am I? A lamb to put me to slaughter when they want to*?” (A8).

Some women believed that they had a CS in their first pregnancy because their doctors did not understand their needs and did not support them; during their second pregnancy, they felt that a female doctor would be more understanding:

“*A woman would understand a woman better”* (A5).

A strong desire to deliver by vaginal birth was described by all mothers. A woman, who gave birth in a public hospital described her experience as follows:

“*I felt the need to … take the situation into my own hands. Therefore, I decided to find an obstetrician who supports and undertakes cases of VBAC*” (A10).

### Seeking information

To fulfil their desperation for clear and tailored to their needs answers, the women sought information about VBAC in a variety of sources besides their consultations with obstetricians. The women sought information on the internet in the form of scientific articles, reports, and newspapers, in VBAC Facebook groups, and in prenatal classes. Some women found the online support groups for VBAC to be a good source of information and support; other women’s successful personal stories made them feel stronger and empowered. Like one of the women said, the injustice she had felt as a result of her previous delivery, led her to make efforts to find people who felt the same way:

“*I started looking for people to talk to. Because... I didn't want to be alone. I didn't want to believe that I'm the only one experiencing this*” (A1).

On the other hand, however, there were women who described their experience with the online support groups as overwhelming; for them, the plethora of information, which was sometimes conflicting, was not particularly helpful.

Prenatal classes were a useful source of information, although the information given in these classes was not specifically related to VBAC practices. Even so, some women considered that it was important for them to attend prenatal classes. They found the prenatal classes extremely useful as they had the opportunity to meet and talk to mothers who had a positive VBAC experience. Some mothers encouraged the women to trust their bodies and suggested that prenatal classes could help them with that.

“*Perhaps it would be a good thing if all mothers had been informed before. Prenatal seminars are useful and offer support.*”

Many women expressed the opinion that a supportive environment was an important contributing factor for a successful VBAC. The women felt they benefited more when they received information from mothers who have had a successful VBAC and they viewed their positive experiences as inspiring examples. These mothers were viewed as idols who share their stories, in order to empower other women and stop obstetric violence:

“*When I realized that there is violence against women... I looked at this idea for VBAC so seriously to protect my daughter from going through the same thing*” (A1).

A woman referred to the absolute trust women have in their doctors, which prevents them from claiming their rights at the time of childbirth and experience the miracle of vaginal childbirth:

“*I was very enthusiastic and I was trying to convey my experience to other mothers, but I realized that women unfortunately have complete confidence in their doctors and they did not search for reliable information*” (A5).

It was not uncommon for the women interviewed in this study to change doctors when they had found a more supportive environment elsewhere, especially in circumstances when they would realize that their obstetrician was giving them biased, wrong or incomplete information from their initial doctor.

### Birth environment (influences)

The women referred to the following factors, which they thought were of vital importance to them regarding their decision-making process and the factors that determined whether the birth environment was supportive or unsupportive for them: a) Father’s involvement, b) Family influences, and c) Interactions with health professionals.

### Father’s involvement

The women felt that the father’s involvement during pregnancy and labour was beneficial towards their lived experience of VBAC, as it helped them reduce their stress levels and that had, overall, a positive impact on their childbirth experience. Some women felt safe knowing that their husband was there for them and supported them during childbirth. Most women described the father's participation in childbirth with gratitude. A woman reported “feeling in love” with her husband at the time of childbirth because of his support:

“*My husband helped me so much. He was with me and I said to him that our daughter was born by the two of us”* (A2).

On the other hand, some women described the father’s influence towards their decision-making process as a negative one and referred to their husband’s anxiety and disagreement for opting for a VBAC as a barrier towards their final decision for opting for VBAC. Especially in cases when the doctor informed them that there would be a risk for rupture of the uterus; some fathers refused to support their wife’s wish to opt for VBAC. A woman described her reaction as follows:

“*I let a couple of weeks pass by and I discussed it with him again and I said to him that I know my body and I want to have a vaginal birth and I believe I can do it*” (A10).

Other women, with similar influences from the father, emphasized that it is important for the mother to make sure that the father of the baby is informed and prepared about VBAC. The women were of the opinion that it could make a difference in the way they supported their wife during her decision-making process and during labour:

*“I attended antenatal classes with my husband and I insisted [for him] to be there with me”* (A3).

Attending antenatal classes was viewed as necessary for husbands/ partners by the women, for gaining the relevant knowledge, understanding more about childbirth and providing their wives with the necessary support, whatever their decision may be. One woman described how she was influenced by her partner:

*“My partner was a 'VBAC baby' and it made me think that this was karmic and made me even more decisive about opting for it (A1)”.*

### Family influences

While the father’s participation in the childbirth experience was essential, support from the woman’s family of origin, especially the mother of the pregnant woman, was as important. The women who had emotional/ psychological support from their families in pursuing a VBAC found this support facilitating towards their decision process. A mother who interpreted her own daughter’s birth through CS as unjust to her at the time, encouraged her daughter to opt for a VBAC for her second pregnancy.

On the other hand, several women referred to the negative comments or the disagreements they had during their discussions with family, about VBAC, as barriers to their decision making. Most women highlighted their mothers’ prejudice and distrust of VBACs. One woman admitted that her mother's negative view of VBACs was *“pulling her back a little bit”* but not enough to affect her final decision: *“my mom thought it was risky*” (A7).

Another woman commented that she wished she had more support from her family:

“*Generally, around me, no one ever understood that this idea bothered me. They kept saying that the most important thing is that you and your baby stay healthy*” (A5).

Another woman expressed her frustration and guilt and described the comments that she received from her family as *“psychological war”* (A1). Her family members advised her to go ahead and have a CS delivery right from the start, rather than going through the pain of labour. She eventually delivered her baby via a CS.

### Interactions with healthcare professionals

During the interviews the women expressed how their interactions with healthcare professionals influenced their decision making and their lived experience of VBAC. During their decision-making process the women explained how their interactions with the doctors and midwives had a significant role in a woman’s choice of birthing mode. A woman explained that her doctor was very supportive and optimistic towards her choice, because when she sent her doctor a message to inform her that she was pregnant, she replied “*Ok perfect, let's go for VBAC*” (A9). In other, similar, cases, the doctors asked the women to create a *“visual birth plan”* and share it with them so they could cooperate towards a positive birth experience. In some cases, midwives informed women of their options, but without directing or guiding their decision-making process, leading the women into making a non-biased, informed choice. Some women expressed positive comments about their doctors’ and midwives’ approach, as they were offered holistic care and they paid attention to their individual needs. Some women felt immense gratitude for their doctor, who did not proceed with any intervention without their consent:

“*She [the obstetrician] gave me the choice. She said to me … If you want, I’m here and I can help you give birth. If you don't want we can wait*” (A5).

Regarding their lived VBAC experiences, some of the women commented on the valuable help and support they had received from midwives during childbirth especially when they felt exhausted, which made their VBAC experience a positive one to remember. A woman who gave birth in a public hospital described how encouraging the midwife was to her. Breathing exercises, walking, using balls, relaxing and showering were all mentioned as useful techniques during labour. Another woman described, whilst smiling, the moment she danced alone in the delivery room with soothing music:

“*But then when the contractions become stronger, after 5-6 cm, they put the music on, I was just dancing, I was completely naked and dancing, in the room”* (A1).

However, such positive experiences were in contrast with the women’s previous childbirth experiences. A woman expressed the frustration she felt during CS both because of the interventions she had and because of inadequate support from the midwife:

“*She told me that I must stay in bed and she checked my cervix dilation all the time, she was an old age lady*” (A8).

### Healing through VBAC

Some mothers explained that after their shocking encounter of giving birth via CS, they wished to attempt VBAC as a way to heal previous trauma and ‘let go’ of the negative experience of a previous childbirth. This theme encompasses the physical, emotional, and psychological aspects of the experience. The women feel that their ‘traumatic birth experience’ has been ‘counteracted’ with a subsequent vaginal birth. Supporting subthemes identified under this theme are (a) Overcoming a negative experience with a positive one, (b) Fulfillment, and (c) Bonding with baby.

### Overcoming a negative experience with a positive one

For many women, the experience of VBAC was one of healing. One woman said that she had a desire to prove that she could deliver vaginally as she felt stigmatized for having a CS.

While their experience with CS was difficult and resulted in painful memories, memories from their VBAC were vivid and made them feel nice, as they hardly faced any problems at all during the process. Overall, women described their VBAC experience as a positive experience, which was psychologically and emotionally beneficial for them.

### Fulfillment

When discussing their VBAC experience, the women expressed feelings of fulfillment. The women’s joy and enthusiasm for their successful VBAC was obvious. Descriptions of their VBAC experience included the words *“euphoria”*, *“spectacular”*, *“incredible”*, *“powerful”*, and *“perfect”*. Some women commented on the experience of intermittent auscultation and the freedom of movement they had during a VBAC procedure, in contrast to the continuous monitoring (i.e. cardiotocography) they had experienced during their CS birth, which they characterized as *“bothersome”*. A woman explained that while she had many vaginal examinations during her first childbirth, the healthcare professionals respected her desire for fewer examinations during VBAC:

“*In my first pregnancy they constantly examined me… vaginally, while during my VBAC they did not, they were very cooperative*” (A9).

The positive impact of VBAC was not limited only to the time of labour and postpartum recovery, but it was a healing experience that brought profound change to the lives of the women after that. A mother said “*it is a victory and a closure of an open wound*” (A1).

The women felt empowered and considered their experience with VBAC to be a success, as it proved to all those, who did not believe they could make it via a vaginal delivery and thus refused to support the woman of her choice, that they were wrong. A woman, who felt proud to have succeeded, stated: *“to have a vaginal birth in Cyprus is an achievement”* (A8), while another woman, whilst conveying an expression of victory with her hand, shouted enthusiastically *“I made it!”* (A10). Other women described their experiences in metaphorical and profoundly creative ways, such as *"I found myself in another universe*" (A2) and “*I was so happy I felt I was shining*” (A5). A woman, after taking a deep breath and crying out of happiness said that: “*the satisfaction is incredible that you made it, that... that you climbed a mountain alone”* (A1). At the same time, the women expressed their gratitude for the people who helped them achieve their goal of having a VBAC, including health professionals, family members, and other women who empowered them through their own experiences, which again, refers back to the theme and the importance of birth environment, mentioned earlier in this article.

Bonding with the baby

The participants described positive, prolonged periods of bonding with their newborns following VBAC. Some mothers explained that after the birth of their baby, their feelings and bonding with their babies were significantly different from their previous experience:

“*After having a CS delivery I saw my baby and could not …. I didn’t know how I felt. With VBAC I loved my baby from the first moment”* (A1).

The women’s testimonies reveal that compared with birth via CS, VBAC results in women feeling better and able to offer their newborn the love and care they need.

## Discussion

In this study, women’s experiences of vaginal childbirth after a previous experience of a caesarean section as a birthing mode, have been explored, for the first time in Cyprus. The findings of this study provide a significant insight into women’s own perspectives of their experiences, using a phenomenological methodology in the context of a qualitative study.

Their experience of a previous cesarian section as a mode of birth was viewed as a highly traumatic experience by all women, which has caused them substantial stress and anxiety in the past. Post-traumatic stress as a result of negative experiences during birth is also described in other studies [27, 28]. The literature reveals that it is not uncommon for women to develop a ‘fear of childbirth’ in countries with low VBAC rates [7–9, 29]. Interestingly, this was not a theme that was identified in this study. On the contrary, the theme of ‘overcoming a negative experience with a positive one’ was repeatedly reflected by the statements of the women interviewed, which stated that they were intrigued and motivated by their previously negative experience of a CS into having a VBAC and made them more assertive to ‘fight’ for their choice of mode of birth. International literature suggests that vaginal birth has a personal meaning for women and in general women are strongly determined to birth vaginally after a CS, contributing to their determination to achieve VBAC [7–9, 14–17]. The women described the ‘fulfilment’ they felt after having a vaginal birth and a sense of ‘healing’ (through VBAC) after their achievement. Although research indicates that most women will submit to the recommendation of a CS by their obstetrician [30], the women in this study did not seem to be affected by their obstetricians’ recommendations. It seems that their previous traumatic experience of a CS has affected and influenced their decision-making process during their subsequent pregnancy. This finding aligns with previous research showing that mothers with negative CS experiences have an increased desire for vaginal birth [31, 32].

From a psychological point of view, the women have described VBAC as an empowering and ‘healing’ experience. These views are consistent with those of Fenwick et al.’s [29] and Akgün and Boz’ s[18] findings, where the participants of the study described the profound impact that VBAC had on their self-perception and confidence as women and mothers, and how it positively affected their ‘bonding with the baby’ and breastfeeding. The women in the current study described VBAC as *“an unreal experience”* which made them feel happy and proud of themselves, in being prepared to claim what they had *“lost”* in a past childbirth. Some women, feeling vindicated by the unfair treatment they had received from the doctor in their previous birth, viewed their VBAC success stories as a personal victory and a redemption from the trauma they had *“deep in their soul”*. Overall, the literature suggests that women who have a vaginal childbirth have positive feelings towards their experience and a strong feeling of pride [33, 34]. In VBAC cases, women feel that they are in control, while empathy and support from health professionals offer them a sense of empowerment and achievement [35]. The support of obstetricians and midwives, that were open to the idea VBAC, was noted to have a significantly positive impact on the VBAC experience and the emotional wellbeing of the participants of this study. Similar studies done in countries with high VBAC rates and a birth culture that promotes VBAC such as Finland, the Netherlands, and Sweden, have shown that women benefit in taking informed decisions about the birthing mode [9]. It becomes imperative, therefore, to empower women and families so that they can reclaim control over their childbirth experiences [32].

Several factors in Cyprus lead to the ‘medicalization of childbirth’ and this theme was quite accentuated in this study. It quite apparent that there is ‘a culture of CSs’ in Cyprus and that is clearly reflected not only through the women’s memories of their doctor’s recommendations, but also from their family influences, as described in the results under the main theme ‘Birth environment’. The women frequently mentioned that it was often difficult to find an obstetrician willing to offer the choice of VBAC. Having a reliable and trustable communication with healthcare professionals, who display supportive attitudes, is vital throughout pregnancy and this is highlighted throughout the literature [7–9, 14]. The role of the midwife in supporting women during pregnancy, providing pregnant women with answers to their questions and guidance, as well as support women in labour, is not valued enough in Cyprus. Midwives in Cyprus find themselves in difficult situations when advocating vaginal childbirth, due to medical domination of the healthcare services [10, 36] and this may be one of the factors that has led to the ‘medicalization of childbirth’ and ‘a culture of CSs’ in Cyprus [37].

‘Preparing for a VBAC’ is a theme that emerged from the women’s descriptions about their numerous efforts to seek proper information during their decision-making process. The women’s descriptions that were related to the theme ‘seeking information’ implied that their obstetrician’s opinions were biased. The women would frequently visit other doctors in their constant search for ‘clear answers’, an experience they described as particularly stressful; such interpretations were constantly reflected under the theme ‘preparing for VBAC’.

‘Lessons learned’ following a previous caesarean delivery, seemed to have influenced the women in becoming determined to seek updated and reliable information on VBAC. A number of online sources (e.g. newspapers and scientific articles) were mentioned to have been useful, but most women found the VBAC support groups on social media particularly helpful in acquiring information and support from other women, who shared their stories on similar experiences. Such interactions were empowering to the women and gave them courage. A global survey by Lagan et al. [38] highlighted the benefits of attending support groups for pregnant women. The women interviewed described how other women’s stories had a positive impact on them, as they reduced stress and isolation, and offered support, reassurance, and facilitated their decision-making process.

### Limitations of the study

The findings of this study cannot be generalized to the whole population of women who have experienced VBAC. The recruitment of participants was implemented through a specific social media platform - a local Facebook VBAC support group - and therefore the sample consisted of women who were members of that particular group and their social networks. Therefore, these women may have had similar characteristics in terms of socio-economic status (e.g. all had access to internet and were university-educated). In addition, these women were self-selected and their views, experiences, and interpretations may not reflect all women's experiences of VBAC in Cyprus. The women interviewed were aged between 28-38 years old, which is only partly representative of the population of women of reproductive capacity. Nonetheless, the in-depth insight into the participants’ accounts can serve as a starting point for further reflection and discussion on the perinatal care in Cyprus and its improvement.

## Conclusions

Vaginal childbirth after a previous caesarean section is a relatively rare practice in Cyprus and there are no recorded statistical and qualitative data of VBAC cases. A woman’s journey to VBAC is often described as a personal victory, as an *“inaccessible mountain”* that the women managed to climb. Giving birth vaginally after CS is often described by women as an empowering experience [7, 9]. While some women expressed gratitude about the strength and support they received from family, support groups and certain healthcare professionals, other women highlighted the *“psychological war”* they had to face by some obstetricians and the lack of supportive attitude when discussing the possibility of a VBAC. The need for accurate and reliable information and guidance during pregnancy and childbirth, as well as the need for respecting women’s right of choice was strongly emphasized throughout the interviews. The findings of this study can inform health professionals on the profound physical and emotional experiences associated with VBAC and encourage them to reassess current practices for improved care.

Local healthcare policy makers can improve the perinatal care offered by requiring perinatal health professionals and obstetricians to become educated, trained, and informed (e.g through multidisciplinary seminars) on the psychological effects of CSs on women, the psychological benefits of VBAC and to offer pregnant women alternative choices of birthing mode (such as TOLAC) [39], in light of the right for women to make informed choices on the delivery of their baby. Evidence-based maternity care requires a balanced understanding of the actual risks of VBAC but also acknowledgement of the woman’s emotional status, needs and perspectives. At the same time, each maternity hospital must ensure that there is a protocol in place for VBAC.

Overall, all the women interviewed for this study described their VBAC experiences in a positive manner and strongly highlighted the need to help other women with their decision-making process and help them face any barriers that may occur because of their choice to deliver their babies vaginally after a previous experience of CS. These women have the right to informed choice and one of the ways to raise awareness about it is through social media, according to the findings of this study. Pregnant women in Cyprus would vastly benefit from the set up of ‘walk in’ clinics, where they will have the opportunity to seek emotional help and counseling by professionals relating to the themes discussed in this paper. These clinics could be staffed by specially trained midwives that can offer support to women through their ‘journey’ of pregnancy and decision-making process and also help them to emotionally cope with past traumatic experience of childbirth. Such implementations will provide ongoing opportunities for quality improvement in the national health system scheme of Cyprus and further opportunities to explore women's experiences of VBAC, as there is a need for balanced, unbiased and evidence-based information on the subject of VBAC. This could facilitate the provision of better quality care in countries with lower rates of VBAC.

Suggestions for further research on the topic include the views of women and couples of reproductive age from different socio-economic backgrounds, as well as the perceptions and experiences of women who opted for VBAC but were not able achieve it. Furthermore, future research could expand to explore the perceptions and experiences of perinatal health care providers in Cyprus on VBAC, such as in the case of Firoozi et al. [40]. The exploration of health care professionals’ knowledge and attitudes towards VABC could be a useful source of information for the health care authorities. Such studies can contribute to our understanding of the reasons behind low rates of VBAC in Cyprus, as well as influence policymaking accordingly.

## Data Availability

The data that support the findings of this study is in Greek but could be available from the authors upon reasonable request and with permission of CK, EH.

## Abbreviations

VBAC: Vaginal Birth after Cesarean
TOLAC: Trial of labor after cesarean
C/S: Cesarean section
ACOG: American College of Obstetricians and Gynecologists
WHO: World Health Organization
GDPR: General Data Protection Regulation

## Footnotes

1 Refers to the areas controlled by the Republic of Cyprus.
